# Supplementary material for: The postcranial skeleton of the erythrosuchid archosauriform Garjainia prima from the Early Triassic of European Russia
Source: R Soc Open Sci. 2020 Dec 2;7(12):201089. doi: 10.1098/rsos.201089 (PMC7813270; doi:10.1098/rsos.201089)
Supplement: Online Supplementary Material [file rsos201089supp1.docx]

**The postcranial skeleton of the erythrosuchid archosauriform *Garjainia prima* from the Early Triassic of European Russia**

**Online Supplementary Material**

Susannah C. R. Maidment^1,2*^, Andrey G. Sennikov^3,4^, Martín D. Ezcurra^2,5^, Emma M. Dunne^2^, David J. Gower^1^, Brandon Hedrick^6^, Luke E. Meade^2^, Thomas J. Raven^1,7^, Dmitriy I. Paschchenko^3^, Richard J. Butler^2^

1. The Natural History Museum, Cromwell Road, London SW7 5BD, United Kingdom
2. School of Geography, Earth and Environmental Sciences, University of Birmingham, Edgbaston, Birmingham, B15 2TT, United Kingdom
3. Borissiak Paleontological Institute RAS, Profsoyuznaya Street 123, Moscow 117647, Russia
4. Institute of Geology and Petroleum Technologies, Kazan Federal University, Kremlyovskaya Street 4, Kazan 420008, Russia
5. Sección Paleontología de Vertebrados, CONICET—Museo Argentino de Ciencias Naturales “Bernardino Rivadavia”, Ángel Gallardo 470 (C1405DJR), Buenos Aires, Argentina
6. Department of Cell Biology and Anatomy, School of Medicine, Louisiana State University Health Sciences Center, New Orleans, LA 70112, USA.
7. School of Environment and Technology, University of Brighton, Lewes Road, Brighton BN2 4GJ, United Kingdom

**Axial skeleton**

**
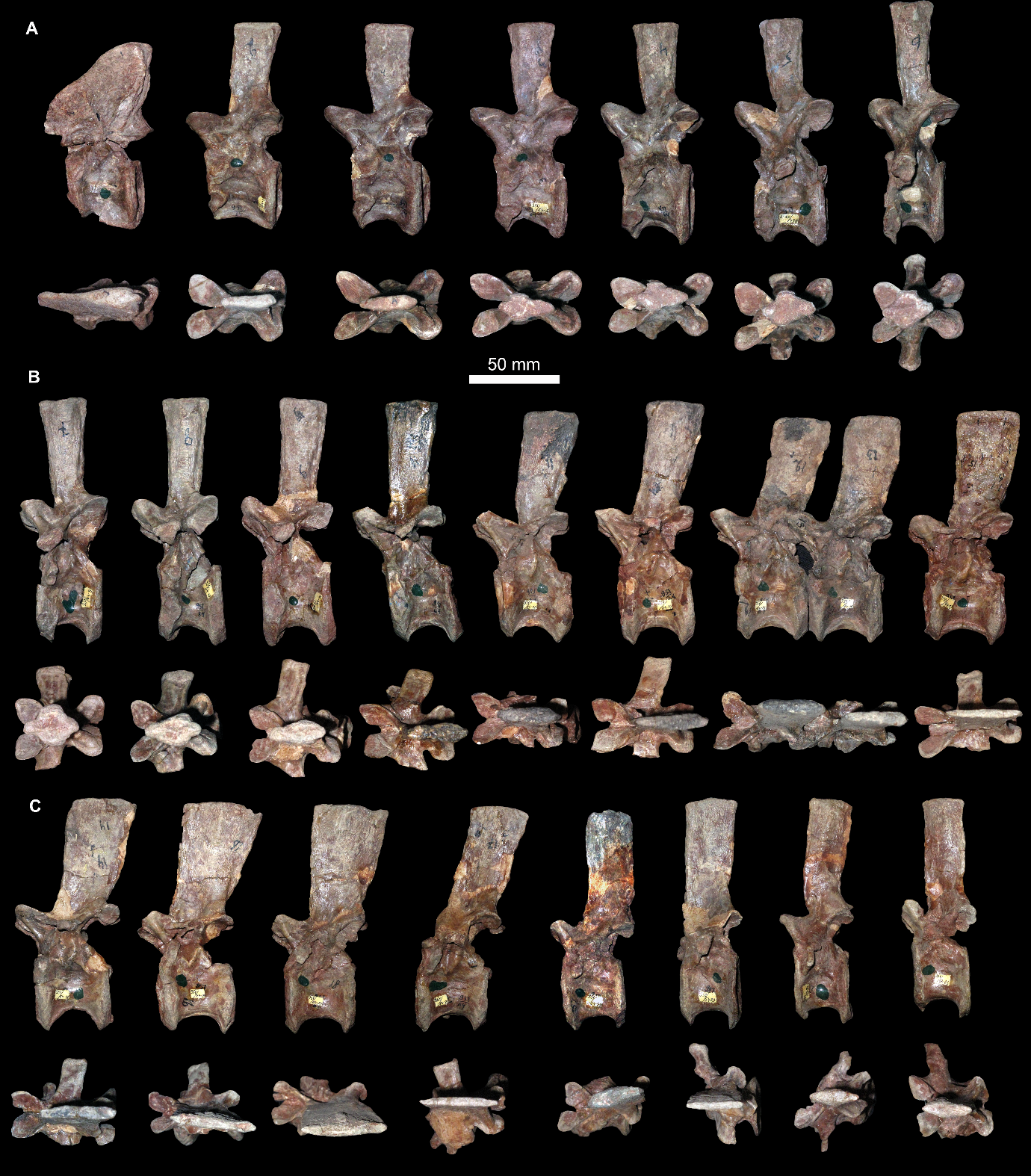
**

**Figure S1**. **Articulating series of presacral vertebrae, PIN 951/64, in left lateral and dorsal views. A**, presacrals 2–8; **B**, presacrals 9–17;  **C**, presacrals 18–25. ­­­Scale bar equal to 50 mm.

The following is a list of referred (non-type) vertebral material of *Garjainia prima* preserved in addition to the articulated series of vertebrae catalogued as PIN 951/64.

PIN 951/35-3: dorsal centrum, ventral margins reconstructed in plaster, missing neural arch and spine.

PIN 951/40-2: anterior caudal vertebra with damaged anterior centrum face, caudal ribs broken, missing most of neural arch and neural spine.

PIN 951/35-8: vertebra from cervico-dorsal transition, c. presacral 8, with three articular facets.

PIN 951/35-2: vertebra from cervico-dorsal transition with three articular facets, missing much of neural arch, spine, distal part of right transverse process.

PIN 951/35-21: vertebra from mid-dorsal region (c. presacral 16–17), missing neural spine.

PIN 951/35-18: vertebra from mid-dorsal region (c. presacral 16–17), missing most of neural spine.

PIN 951/35-20: posterior dorsal vertebra lacking left transverse process.

PIN 951/35-23: posterior dorsal vertebra lacking most of neural spine and right transverse process.

PIN 951/40-1: anterior caudal vertebra lacking left caudal rib and distal end of right caudal rib.

PIN 951/35-4: vertebra from cervico-dorsal transition with three articular facets, almost complete.

PIN 951/35-10: large mid-dorsal vertebra, missing right transverse process.

PIN 951/35-16: posterior dorsal vertebra, missing transverse processes and most of neural spine.

PIN 951/35-22: posterior presacral or anterior caudal vertebra, missing caudal ribs and neural spine.

PIN 951/35-7: large mid-dorsal vertebra, almost complete.

PIN 951/35-11: large mid-dorsal vertebra, mostly complete, missing right transverse process.

PIN 951/40-4: anterior caudal vertebra missing neural spine, most of neural arch except right prezygapophysis, and caudal ribs.

PIN 951/37-1: sacral vertebra 1, largely complete but missing left sacral rib.

PIN 951/25-4: anterior cervical vertebra, almost complete.

PIN 951/40-6: caudal vertebra, missing prezygapophyses and caudal ribs.

PIN 951/25-5: anterior cervical vertebra, complete, with cervical ribs glued into articulation.

PIN 951/35-9: anterior to mid-dorsal vertebra, lacking neural spine and right transverse process.

PIN 951/35-1: vertebra from cervico-dorsal transition with three articular facets, almost complete.

PIN 951/40-3: anterior caudal vertebra, lacking neural spine, most of neural arch, caudal ribs.

PIN 952/35-13: anterior dorsal vertebra with three rib articulation facets, probably equivalent to presacral 12. Largely complete.

PIN 952/35-14: mid-dorsal vertebra. Largely complete.

PIN 952/35-12: mid-dorsal vertebra. Largely complete.

PIN 952/35-17: mid-dorsal vertebra, missing neural spine and most of transverse processes.

PIN 951/25-6: anterior cervical vertebra, complete.

PIN 952/35-6: mid-dorsal vertebra. Largely complete.

PIN 952/35-15: posterior dorsal vertebra. Largely complete.

PIN 952/35-19: posterior dorsal vertebra. Largely complete.

Various isolated fragments of neural spines are present.


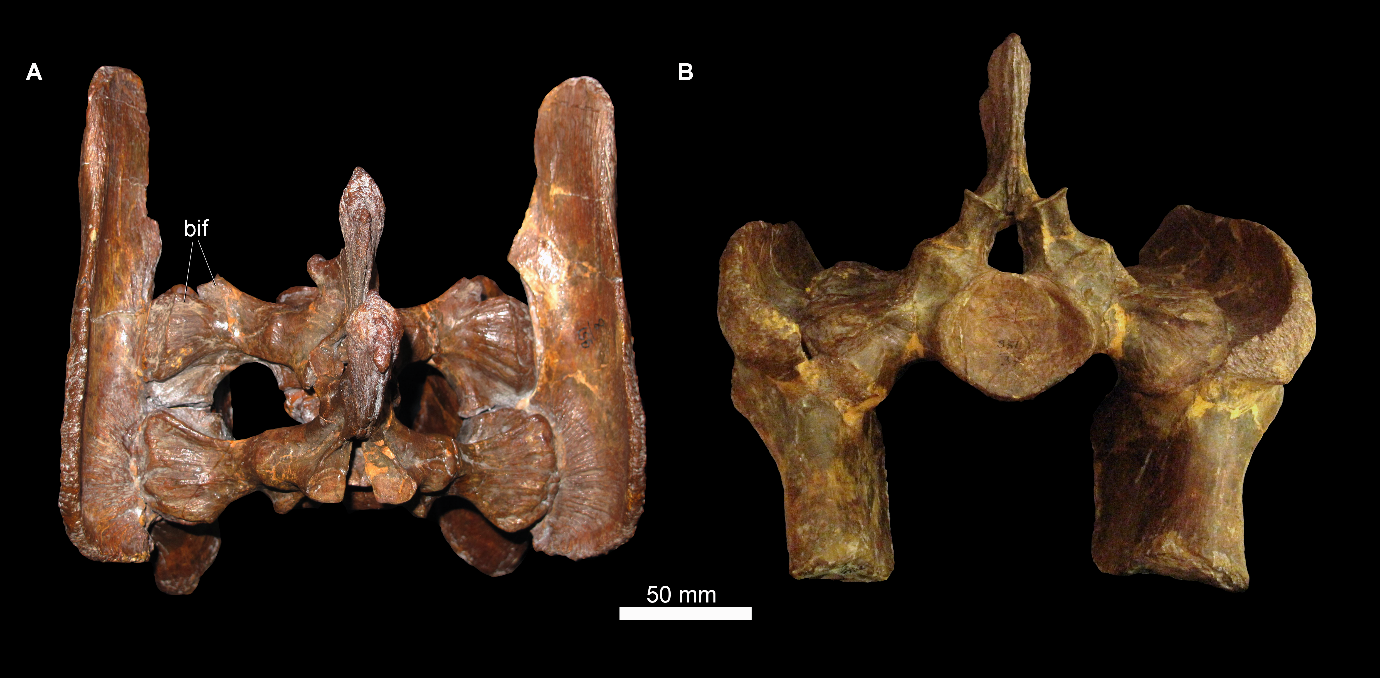


**Figure S2. PIN 951/37-1, 2, articulating sacral vertebrae 1 and 2 and ilia. A**, dorsal view; **B**, anterior view**. bif**, bifurcation of distal end of sacral rib 2. Scale bar equal to 50 mm.

The following table lists measurements for the articulated vertebral series PIN 951/64, described in the manuscript.

| **Vertebra number** | **Anteroposterior length of the centrum** | **Dorsoventral height of the anterior face of the centrum** | **Transverse width of the anterior face of the centrum** | **Dorsoventral height of the posterior face of the centrum** | **Transverse width of the posterior face of the centrum** | **Total height of the vertebra from base of centrum to tip of neural spine** | **Height of neural spine from the top of the postzygpophyses to tip of neural spine** |
| --- | --- | --- | --- | --- | --- | --- | --- |
| 2 | 36 | 32 | 24 | 24 | 19 | 106 | 65 |
| 3 | 38 | 32 | 25 | 34 | 23 | 115 | 50 |
| 4 | 36 | 33 | 24 | 34 | 26 | 118 | 53 |
| 5 | 38 | 29 | 23 | 38 | 24 | 121 | 53 |
| 6 | 38 | 36 | 27 | 38 | 24 | 126 | 55 |
| 7 | 36 | 40 | 24 | 39 | 23 | 131 | 54 |
| 8 | 32 | 39 | 26 | 40 | 27 | 132 | 55 |
| 9 | 33 | 33 | 40 | 35* | 26 | 132 | 58 |
| 10 | 36 | 37 | 24 | 39 | 23 | 133 | 59 |
| 11 | 38 | 37 | 25 | 34* | 20* | 133 | 63 |
| 12 | 36 | 34 | 23 | 35 | 21 | 130 | 60 |
| 13 | 38 | 32 | 23 | 32 | 19 | 126 | 61 |
| 14 | 37 | 34 | 19 | 31 | 21 | 129 | 62 |
| 15 | 37 | 32 | 23 | NA | NA | 125 | 60 |
| 16 | 41 | NA | NA | 27 | 21 | 125 | 62 |
| 17 | 41 | 28 | 17 | 27 | 20 | 125 | 63 |
| 18 | 42 | 27 | 20 | 27 | 20 | 124 | 59 |
| 19 | 38 | 30 | 19 | 27 | 19 | 123 | 61 |
| 20 | 37 | 27 | 19 | 30 | 21 | 122 | 61 |
| 21 | 39 | 30 | 15 | 29 | 19 | 122 | 63 |
| 22 | 32 | 32* | 23* | 32* | 27 | 119 | 66 |
| 23 | 30 | 31 | 27 | 30 | 29 | 118 | 58 |
| 24 | 29 | 31 | 28 | 34* | 27 | 118 | 59 |
| 25 | 28 | 31 | 30 | 32 | 30 | 113 | 60 |
| 26 | 36 | 33 | 44 | 25 | 30 | NA | NA |
| 27 | 34 | 23 | 32 | 22* | 29* | NA | NA |
| 28 | 24 | 29 | 28 | NA | NA | NA | NA |
| 29 | 31 | 29* | 20* | 26 | 21 | 97 | 47 |
| 30 | 25 | 28 | 26 | 26* | 21* | NA | NA |
| 31 | 27 | 26 | 16* | 26 | 18* | NA | NA |

*****Slightly incomplete or damaged

**Appendicular skeleton**

The following lists all referred (non-type) appendicular material of *Garjainia prima* in the PIN collections and provides measurements of this material.

**Coracoids**

Two complete scapulocoracoids are similar in size and preservation and likely belong to the same individual. The right is **PIN 951/4-1-1**, and the left is **951/4-1-2**. The clavicle is also preserved fused to the dorsal margin of the coracoid and scapula in the right element but not on the left, and this appears to be the only scapulocoracoid in the collection that preserves the clavicle as well. **PIN 951/3** is a large left proximal scapula and is associated with (glued to) a partial coracoid, with the coracoid missing its ventral margin. **PIN 951/2-1-1 and 2-1-2** are two coracoids that are unfused to scapulae and may be from the same individual. **PIN 951/2-2-2** is a large right coracoid. A fragment of another very small coracoid (PIN 951/4-4) is present.

| **Specimen number** | **Anteroposterior length (mm)** | **Dorsoventral height (mm)** |
| --- | --- | --- |
| 951/2-2-2 | 139.7 | 102.9 |
| 951/2-1-1 | 116.8 | 78.7 |
| 951/3 | 135.1 | Incomplete |
| 951/2-1-2 | Incomplete | 80.8 |
| 951/4-1-1 | 128.7 | 78.0 |
| 951/4-1-2 | Incomplete | 79.9 |

**Scapulae**

There are seven complete and four partial scapulae in the collection. **PIN 951/3** is a proximal scapula with articulating coracoid. **PIN 951/4-1-1** and **951/4-1-2** are a pair of articulating scapulocoracoids. The remaining scapulae are not articulated to coracoids (although some may be associated) and comprise: **PIN 951/4-2**, a large left element, **PIN 951/7-1-2**, a medium left element, **PIN 951/7-1-1** a small left element, **PIN 951/2-2-1**, a large right element, which based on size and morphology may be from the same individual as **PIN 951/4-2**, and **PIN 7-1-1,** a smaller right element, which based on size, preservation and morphology is probably from the same individual as **PIN 951/7-1-2**. Based on size alone, the left and right coracoids **PIN 951/2-1-1** and **PIN 951/2-1-2** may belong to these scapulae. Based on size and preservation, the scapula **PIN 951/2-2-1** and the coracoid of **951/2-2-2** are likely from the same individual. **PIN 951/70** is a distal end of a scapula from the same locality but collected at a later date (1974). **PIN 951/56** is a partial distal end of a scapula of a small specimen. Based on these assumptions, seven individuals are represented by scapulocoracoid material in the collection.

| **Specimen number** | **Dorsoventral height (mm)** | **Dorsal end width (mm)** | **Ventral end width (mm)** | **Transverse thickness of glenoid (mm)** |
| --- | --- | --- | --- | --- |
| PIN 951/4-3 | 160.0 | Incomplete | 79.0 | 21.4 |
| PIN 951/7-1-2 | 188.0 | Incomplete | 97.9 | 41.3 |
| PIN 951/4-2 | 225.0 | Incomplete | 116.7 | 62.6 |
| PIN 951/2-2-1 | 228.0 | 137.0 | 111.9 | 51.7 |
| PIN 951/7-1-1 | 190.0 | Incomplete | 94.5 | 30.0 |
| PIN 951/4-1-1 | 208 | Incomplete | 103.1 | 47.7 |
| PIN 951/4-1-2 | 207 | 97.2 | 109.4 | 45.8 |

**Humeri**

There are six partial or complete humeri in the collections. These are: **PIN 951/36-5**, the proximal end of a very small right humerus; **PIN 951/62**, a small right humerus; **PIN 951/36-4,** a right humerus; **PIN 951/36-3,** a small left humerus, which based on size may belong to the same individual as **951/36-4**; **PIN 951/36-2,** a large right humerus, and **PIN 951/36-1**, and a large left humerus**.**

| **Specimen number** | **Humerus length (mm)** | **Maximum width, proximal end (mm)** | **Maximum width, distal end (mm)** | **Minimum shaft circumference (mm)** |
| --- | --- | --- | --- | --- |
| PIN 951/62 | 153.1 | 85.7 | 86.9 | 83 |
| PIN 951/36-3 | 177.0 | 107.4 | 89.4 | 84 |
| PIN 951/36-4 | 165.0 | 93.0 | 88.8 | 83 |
| PIN 951/36-2 | Incomplete | 106.8 | Incomplete | 94 |
| PIN 951/36-1 | 193.0 | 123 | 98.7 | 107 |

**Ulnae**

Two right ulnae of slightly different lengths are present. **PIN 951/38-1** is the larger element and is better preserved; **PIN 951/38-2** is slightly smaller, and is glued to a radius (**PIN 951/132**) from an old mount.

| **Specimen number** | **Length (mm)** | **Maximum proximal width (mm)** | **Maximum distal width (mm)** |
| --- | --- | --- | --- |
| PIN 951/38-1 | 154.1 | 53.1 | 36.7 |
| PIN 951/38-2 | 141.6 | 53.2 | 28.5 |

**Radii**

Two right radii are present. **PIN 951/132** is a larger specimen and is glued to an ulna for an old mount. It was mounted upside down. **PIN 951/131** is smaller.

| **Specimen number** | **Length (mm)** | **Proximal width (mm)** | **Distal width (mm)** |
| --- | --- | --- | --- |
| PIN 951/132 | 136.6 | 48.6 | 32.9 |
| PIN 951/131 | 112.2 | 40.1 | 28.8 |

**Ilia**

In the collection there are seven ilia: **PIN 951/49** and **PIN 951/48** a pair of ilia from a single individual; **PIN 951/8-2-1 and 8-2-2**, a pair of ilia from a single individual; **PIN 951/8-1-1 and 8-1**-2, a pair of ilia from a single individual; and **PIN 951/8-3,** a single ilium missing its postacetabular process.

| **Specimen number** | **Preacetab-postacetab process length (mm)** | **Postacetabular length (mm)** | **Maximum height (mm)** |
| --- | --- | --- | --- |
| PIN 951/49 left | Incomplete | 76 | Incomplete |
| PIN 951/48 right | 138 | 80 | 107.6 |
| PIN 951/8-1-2 left | 156 | 83 | 125 |
| PIN 951/8-1-1 right | 158.7 | 85 | 122 |
| PIN 951/8-2-1 right | 134 | 69.6 | 122.5 |
| PIN 951/8-2-2 left | 138.6 | 68.3 | 118.6 |
| PIN 951/8-3 incomplete | Incomplete | Incomplete | 115 |

**Pubes**

Six pubes and an additional iliac peduncle are present. **PIN 951/130** is an isolated iliac peduncle. **PIN 951/5-1 and 5-2** are a pair of pubes which were part of the mount; **PIN 951/5-3** is a left pubis; **PIN 951/5-4** is a left pubis; **PIN 951/53** is a partial right pubis that may be the same individual as **PIN 951/52**; **PIN 951/52** is a partial left pubis.

| **Specimen number** | **Maximum length (mm)** | **Maximum width (mm)** |
| --- | --- | --- |
| PIN 951/5-1 right (mounted) | 127.4 | 65.4 |
| PIN 951/5-2 left (mounted) | 134 | 75.5 |
| PIN 951/5-4 left | 123.6 | Incomplete |
| PIN 951/5-3 left | 119.1 | Incomplete |
| PIN 951/53 right | 113.7 | Incomplete |
| PIN 951/52 left | 109.5 | Incomplete |

**Ischia**

Six ischia, corresponding to three pairs, are present. Two ischia are glued together for the mount. The left is labelled as **PIN 951/1-1-2**, the right is labelled as **PIN 951/1-1-1**. These are the largest. A smaller pair are **PIN 951/1-2-1 and 1-2-2**. The final pair are **PIN 951/50** (right) and **PIN 951/51** (left). These articulate with ilia and pubis with sequential numbers and are clearly from a single individual.

| **Specimen number** | **Length (mm)** |
| --- | --- |
| PIN 951/51 | 150.6 |
| PIN 951/50 | Incomplete |
| PIN 951/1-1-2 (mounted) left | 185 |
| PIN 951/1-1-1 (mounted) right | 190 |
| PIN 951/1-2-1 left | 157.7 |
| PIN 951/1-2-2 right | 160.4 |

**
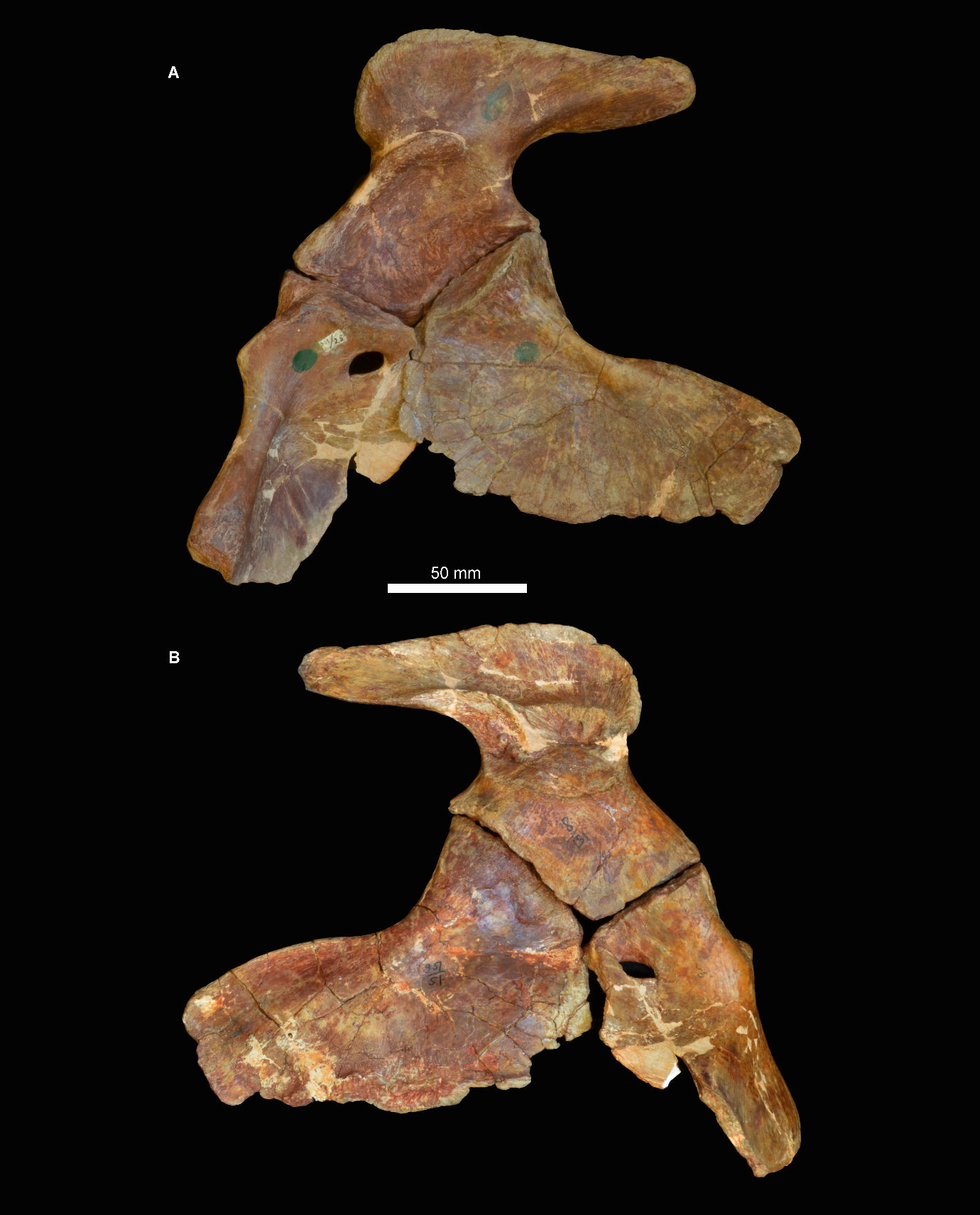
**

**Figure S3. Articulating pelvis, PIN 951/8-2-2, 951/51 and 951/5-3. A**, left lateral and **B**, left medial views. Scale bar equal to 50 mm.

**Femora**

There are two femora and an additional distal end in the collection. A large right femur **PIN 951/61-1**, a small left femur **PIN 951/61-2**, and the distal end **PIN 951/6-1**.

| **Specimen number** | **Length (mm)** | **Prox width (mm)** | **Dist width (mm)** | **Minimum circumference (mm)** |
| --- | --- | --- | --- | --- |
| PIN 951/61-1 | 243 | 71 | 87 | 106 |
| PIN 951/61-2 | 211 | 57 | Incomplete | 84 |

**Tibiae**

There are two tibiae in the collections. Identification is difficult but **PIN 951/43-1** is a right tibia and **PIN 951/43-2** is a smaller left element with a fibula glued to it. The fibula is glued in the wrong place.

| **Specimen number** | **Length (mm)** | **Proximal width (mm)** | **Distal width (mm)** |
| --- | --- | --- | --- |
| PIN 951/43-1 | 217 | 74 | 48 |
| PIN 951/43-2 | 200 | 65 | 45 |

**Fibulae**

There are two fibulae in the collections. One is glued to a tibia in the wrong orientation, is a right element and has the number **PIN 951/124**. The other is larger, right element, and is **PIN 915/126**.

| **Specimen number** | **Length (mm)** | **Proximal width (mm)** | **Distal width (mm)** |
| --- | --- | --- | --- |
| PIN 951/126 | 225 | 35 | 39 |
| PIN 951/124 | 197 | 32 | Incomplete |

**Metatarsals**

| **Specimen number** | **Length (mm)** | **Proximal width (mm)** | **Minimum transverse width of shaft (mm)** | **Distal width (mm)** |
| --- | --- | --- | --- | --- |
| PIN 951/86 | 74 | 31 | 11 | 23 |
| PIN 951/84 | 68 | 28 | 10 | 21 |
| PIN 951/87 | 70 | 27 | 22 | 23 |
| PIN 951/85 | 59 | 29 | 18 | 18 |
| PIN 951/88 | 51 | 22 | 10 | 18 |
| PIN 951/111 | 49 | 31 | 11 | 19 |
| PIN 951/109 | 40 | 31 | 12 | 20 |
| PIN 951/112 | 41 | 28 | 15 | 18 |
| PIN 951/110 | 35 | 26 | 14 | 16 |

**Phalanges**

| **Specimen number** | **Length (mm)** | **Proximal width (mm)** | **Minimum transverse width of shaft (mm)** | **Distal width (mm)** |
| --- | --- | --- | --- | --- |
| PIN 951/115 | 29 | 19 | 10 | 17 |
| PIN 951/113 | 22 | 16 | 9 | 13 |
| PIN 951/114 | 20 | 15 | 9 | 13 |
| PIN 951/89 | - | 21 | - | - |
| PIN 951/90 | 30 | 23 | 15 | 17 |
| PIN 951/91 | 29 | 20 | 11 | 15 |
| PIN 951/92 | 30 | 19 | 10 | 15 |
| PIN 951/93 | 27 | 21 | 14 | 17 |
| PIN 951/94 | 24 | 19 | 10 | 16 |
| PIN 951/95 | 24 | 18 | 10 | 16 |
| PIN 951/96 | 30 | 16 | 9 | 13 |
| PIN 951/97 | 22 | 17 | 9 | 12 |
| PIN 951/98 | 19 | 15 | 9 | 15 |
| PIN 951/99 | 17 | 16 | 10 | 14 |
| PIN 951/101 | 16 | 13 | 8 | 11 |
| PIN 951/102 | 18 | 13 | 9 | 11 |
| PIN 951/103 | 15 | 13 | 9 | 11 |
| PIN 951/104 | 23 | 8 | 8 | 10 |
| PIN 951/105 | 14 | 9 | 5 | 5 |

**Ungual phalanges**

| **Specimen number** | **Length (mm)** |
| --- | --- |
| PIN 951/21 | 32 |
| PIN 951/80 | 18 |
| PIN 951/79 | 25 |
| PIN 951/78 | 22 |

**Phylogenetic Analysis**

Characters modified from the previous version of the phylogenetic data matrix.

393. Scapula, lateral tuber on the posterior edge, just dorsal of the glenoid fossa: absent (0); present (1) (Nesbitt, 2011: 219; Pritchard et al., 2015: 146; Ezcurra, 2016: 393). Butler et al. (2019b) added a third state to this character to describe the presence of a ridge around the level of maximum anteroposterior compression of the scapular blade in *Guchengosuchus shiguaiensis*, *Erythrosuchus africanus* and *Shansisuchus shansisuchus*. However, here we recognized the presence of both a tuber immediately dorsal to the glenoid fossa and a ridge on the posterior surface of the scapular blade in *Garjainia prima*. The former feature is interpreted as a correlate of the m. scapulotriceps, whereas the ridge on the blade is a correlate of the m. scapulohumeralis caudalis based on the anatomy of extant archosaurs. As a result, both features are not homologous and we deleted the third state of character 393. The character 602 of the phylogenetic data matrix describes the presence of a distinct, longitudinal ridge on the posterior surface of the scapular blade (Nesbitt et al., 2017) and we interpret it the correlate of the m. scapulohumeralis caudalis. As a consequence, the above mentioned erythrosuchids that were scored as state 2 of character 393 and rescored here in character 602.

Character scorings modified from the previous version of the phylogenetic data matrix.

*Chalishevia cothurnata* (changes following Butler et al. 2019c)

Character 37: changed from (?) to (1).

Character 286, 287, 289, 312, 317, 319, 334, 336, 337, 340, 351, 353, 355, 360, 657, and 658: changed from (0) to (?).

Character 310, 311, 315, 316, 332, 354, 660, and 681: changed from (1) to (?).

*Erythrosuchus africanus*

Character 393: changed from (2) to (0) (see above).

Character 602: changed from (0) to (1) (see above).

*Garjainia prima* (changes following Butler et al. 2019a and this paper)

Character 171: changed from (1/2) to (2).

Character 191 and 193: changed from (?) to (0/1).

Character 320: changed from (1) to (2).

Character 322: changed from (1) to (-).

Character 112, 159, 276, 307, 326, 346, 366, 376, 380, 570, 586, 587, 599, 600, 601, and 665: changed from (?) to (0).

Character 345: changed from (-) to (1).

Character 348, 377, 435, 436, 458, 484, 489, 490, 530, and 660: changed from (?) to (1).

Character 349: changed from (?) to (1/2).

Character 351: changed from (0) to (0&1).

Character 365: changed from (-) to (2).

Character 373, 375, 393, 475, and 661: changed from (0) to (1).

Character 374: changed from (-) to (?).

Character 585: changed from (?) to (0&1).

Character 602: changed from (?) to (1) (see above).

Character 172 and 589: changed from (?) to (-).

*Guchengosuchus shiguaiensis*

Character 393: changed from (2) to (0) (see above).

Character 602: changed from (?) to (1) (see above).

*Shansisuchus shansisuchus* (changes following Butler et al. 2019c)

Character 37: changed from (0&1) to (1).

Character 68: changed from (0) to (2) based on Wang et al. (2013: fig. 2).

Character 393: changed from (2) to (0) (see above).

Character 602: changed from (?) to (1) (see above).
